# Solvent Free, Microwave Assisted Conversion of Aldehydes into Nitriles and Oximes in the Presence of NH_2_OH·HCl and TiO_2_

**DOI:** 10.3390/molecules15010094

**Published:** 2009-12-29

**Authors:** Lucas Villas-Boas Hoelz, Biank Tomaz Gonçalves, José Celestino Barros, Joaquim Fernando Mendes da Silva

**Affiliations:** Departamento de Química Orgânica, Instituto de Química, Universidade Federal do Rio de Janeiro, Av. Athos da Silveira Ramos 149, CT Bloco A, Cidade Universitária, Rio de Janeiro, RJ 21941-909, RJ, Brazil

**Keywords:** titanium dioxide, aldehydes, nitriles, oximes, microwave irradiation

## Abstract

Aromatic aldehydes bearing electron-donating groups are easily converted into their respective nitriles using NH_2_OH·HCl and TiO_2_ under microwave irradiation, while those bearing an electron-withdrawing group give the corresponding oximes.

## 1. Introduction

Titanium dioxide (TiO_2_) has attracted the attention of synthetic organic chemists due to its application as a heterogeneous catalyst and as a photocatalyst. In addition, TiO_2_ is inexpensive, non-toxic and safe [1]. TiO_2_ may complex with the carbonyl oxygen atoms of aldehydes and ketones, thus activating them for nucleophilic attack [2]. Sharghi and Sarvari converted aldehydes and ketones into the respective amides in the presence of NH_2_OH·HCl and TiO_2_ in a one-pot, solvent free procedure, but with long reaction times [3]. Aldehydes may also be converted into their respective nitriles in good yields and short reaction times using NH_2_OH·HCl under microwave irradiation using, however, the toxic *N*-methyl-2-pyrrolidinone (NMP) as solvent [4]. In the two cases, the mechanism involves oxime generation, but while the amides are obtained through Beckmann rearrangement, the nitriles are generated through dehydration.

The transformation of aldehydes into nitriles is a highly valued reaction, due to the versatility of the latter as starting materials in organic synthesis [8]. Although several methods are known for the transformation of aldehydes to their corresponding nitriles [4,6,7,8,9,10,11,12,13], many drawbacks such as low yields, harsh reaction conditions and tedious work-up procedures limit the use of some of these procedures. Furthermore, some of them use corrosive, toxic, expensive or commercially unavailable reagents [2,4].

Oximes, another important class of aldehyde derivatives, also have useful applications in organic synthesis and in analytical chemistry. These compounds, besides being intermediates for isoxazoline [14] and *N*-hydroxylamino nitrile synthesis [15], also have a potential use in perfumery industry due to their higher stability when compared to their corresponding aldehydes, while retaining a pleasant odour [16].

Based on these facts, we decided to evaluate the conversion of aldehydes into oximes and then into nitriles in the presence of NH_2_OH·HCl and TiO_2_ under microwave irradiation, in order to favor dehydratation over Beckman rearrangement of the intermediate oxime. Product formation was followed by GC-MS and the spectroscopic data of products were compared to those available in the literature.

## 2. Results and Discussion

Since we have been interested in the synthetic and medicinal usefulness of vanillonitrile derivatives, we started our research studying the effects of time and irradiation potency on the conversion of vanillin to its oxime and nitrile under microwave irradiation. The results obtained are summarized in Table 1.

**Table 1:** Time and Potency Effects on the Conversion of Vanillin into Vanillonitrile and Vanillin Oxime.

Entry	Time (min)	Power (W)	% Nitrile	% Oxime	% Others
1	0,5	100	60	40	0
2	1	100	75	25	0
3	5	100	83	4	13
4	1	200	53	34	13
5	1	300	56	29	15
6	2	300	62	20	18

*Conditions*: 1 mmol vanillin, 5 mmol TiO_2_, 5 mmol NH_2_OH·HCl.

The results show that the best conversion rate was obtained by irradiating the solid mixture for five minutes at 100 W (entry 3). Increasing the power (entries 4-6) led to higher yields of undesired products (such as the amide and degradation products), while reduced reaction times furnished higher amounts of the oxime (entries 1-2).

It can also be observed that increasing reaction time from one to five minutes (entries 2 and 3) enhanced vanillonitrile yields, as well as the amounts of undesired products, and thus longer reaction times were not evaluated. For both reaction times no starting material could be detected.

We have also explored the effects of the amounts of TiO_2_ and NH_2_OH·HCl in the reaction mixture on the yield of the nitrile. Reducing the amounts of either of these reagents from 5 mmols to 2.5 mmols led to incomplete conversion of the aldehyde and, at the same time, to enhanced yields of undesired products (results not shown). Thus, after determining the best reaction conditions, we applied them to other aromatic aldehydes, as described in Table 2.

**Table 2:** Conversion of Aldehydes into Nitriles and Oximes in the Presence of NH_2_OH·HCl and TiO_2_ under Microwave Irradiation.

Entry	Aldehyde	% Aldehyde	% Nitrile	% Oxime	% Others
1	Ethylvanillin	0	83	5	12
2	4-Dimethylaminobenzaldehyde	0	84	4	12
3	4-Hydroxybenzaldehyde	0	81	10	9
4	2-Pyrrolecarboxaldehyde	0	77	22	0
5	4-Benzyloxyvanillin	0	82	7	11
6	2-Nitrobenzaldehyde	5	15	80	0
7	4-Nitrobenzaldehyde	2	17	81	0
8	4-Bromobenzaldehyde	0	20	80	0

*Conditions*: 1 mmol aldehyde, 5 mmol TiO_2_, 5 mmol NH_2_OH.HCl, 5 min, 100 W.

**Scheme 1:**
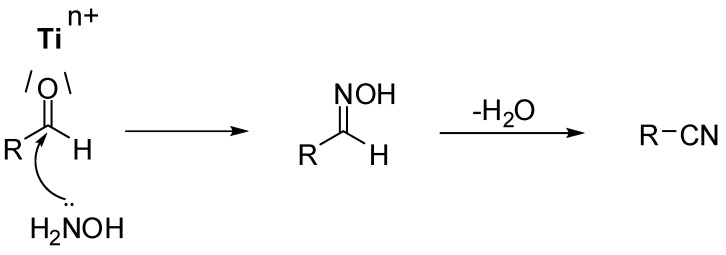
Proposed sequence of transformations during TiO_2_ promoted conversion of aldehydes into nitriles.

The aldehydes bearing electron donating groups were readily converted into the corresponding nitriles (entries 1-5, Table 2) in satisfactory conversion rates (77–84%), whereas substrates bearing electron withdrawing groups (entries 6-8, Table 2) afforded the respective oximes, along with significant amounts of the respective nitriles. In fact, it was previously observed that substrates bearing those groups require longer reaction times for total conversion into nitriles [17]. We further explored the effects of increasing power and reaction times in the conversion of 4-nitrobenzaldehyde to 4-nitro-benzonitrile, but no change in product ratios was observed. GC-MS monitoring supports a two step mechanistic proposal for these reactions (Scheme 1), where the conversion of the aldehyde into the respective nitrile occurs at the expense of the oxime through acid catalysed dehydration.

**Figure 2:**
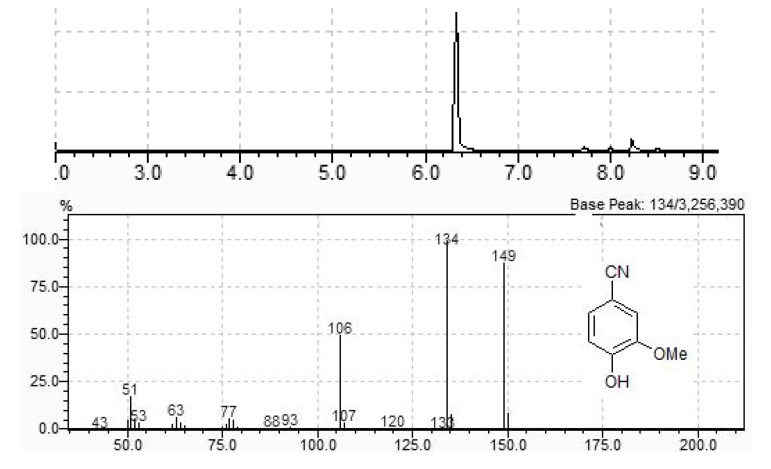
GC chromatogram of scaled up conversion of vanillin into vanillonitrile and MS of the major compound (t_R_ = 6.33 min).

In order to test this hypothesis, we performed the reaction between vanillin and hydroxylamine hydrochloride in the absence of TiO_2_ (100 W, 5 min). The HCl liberated during oxime formation promoted dehydration, but with reduced efficiency when compared to titanium oxide (44% vanillonitrile, 27% oxime, 29% other products). Addition of *p*-toluenesulfonic acid led to a complex mixture of products, while the addition of NaOH or Na_2_CO_3_, either in the presence or absence of TiO_2_ yielded exclusively the oxime with 100% conversion rate. The same occurred with the other aldehydes, what makes this procedure a useful method for rapid, solvent free, microwave assisted synthesis of aldoximes. The reaction can be easily scaled-up, and the synthesis of vanillonitrile using a 4-fold amount of all the reagents led to a 92% conversion to vanillonitrile, which could be purified by flash column chromatography, yielding pure product in 85% isolated yield (Figure 2).

## 3. Experimental

### 3.1. General

The HRGC/MS analyses were carried out on a Shidmadzu QP-2010 operating in electron ionization mode at 70 eV. Helium was used as carrier gas and the injection split ratio was 1:100. Separation was achieved on DB-5 capillary column (30 m × 0.25 mm × 0.25 μm) using the following temperature program: 1 min at 60 °C, 20 ºC/min until 260ºC, then 10 min at 260 ºC. Ion source and injection temperature were at 260 ºC. ^1^H-NMR spectrum of vanillonitrile was recorded on a Bruker HC200 spectrometer, using CDCl_3_ as solvent. The FTIR spectrum was recorded on a Nicolet 550 spectrometer (KBr pellets).

### 3.2. Microwave assisted conversion of aldehydes into nitriles and oximes

A 25 mL reaction flask was charged with a ground mixture of an aldehyde (1 mmol), TiO_2_ (5 mmol, 0.4 g) and hydroxylamine hydrochloride (5 mmol, 0.35 g). The reaction mixture was placed at a CEM Discovery microwave reactor (power-time method) and irradiated at the power settings and reaction times specified in Table 1 and Table 2, with air-cooling and magnetic stirring for the duration of the reaction. After cooling to room temperature, the reaction mixture was resuspended in CH_2_Cl_2_ (10 mL), filtered and a sample submitted to CG/MS analysis with the following results: *3-Ethoxy-4-hydroxybenzonitrile:* t_R_ = 6.92 min. M^+^ = 33.7 (71%). *4-Dimethylaminobenzonitrile:* t_R_ = 7.69 min. M^+^ = 146 (67%). *4-Hydroxybenzonitrile:* t_R_ = 5.94 min. M^+^ = 119 (100%). *2-Pyrrolecarbonitrile:* t_R_ = 3.12 min. M^+^ = 92 (100%). *4-Benzyloxy-3-methoxybenzonitrile:* t_R_ = 7.52 min. M^+^ = 239 (100%). *2‑Nitrobenzonitrile:* t_R_ = 6.09 min. M^+^ = 148 (31%). *4-Nitrobenzonitrile:* t_R_ = 5.39 min. M^+^ = 148 (31%). *4-Bromobenzonitrile:* t_R_ = 6.23 min. M^+^ = 181 (90%), 183 (86%).

### 3.3. Scale-up preparation of vanillonitrile

Vanillin (0.6 g, 4 mmol), TiO_2_ (1.6 g, 20 mmol) and NH_2_OH·HCl (1.4 g, 20 mmol) were ground in a mortar and the mixture was transferred to a 125 mL reaction flask. The reaction mixture was placed at a CEM Discovery microwave reactor (power-time method) and irradiated at 100 W for 5 minutes, with air-cooling and magnetic stirring for the duration of the experiment. After cooling to room temperature, the reaction mixture was resuspended in ethanol (10 mL), filtered and then purified by flash column chromatography using silica gel as adsorbent and hexane-ethyl acetate (7:3) as eluant, yielding pure vanillonitrile in 85% yield, m.p. = 84–87 °C (lit. 85–87 °C); ^1^H-NMR δ ppm 3,93 (s, 3H, OC**H**_3_), 6,20 (bs, 1H, O**H**), 6,94 (d, 1H, C_5_-**H**), 7,02 (s, 1H, C_2_-**H**), 7,35 (d, 1 H, C_6_-**H**); FTIR υ cm^-1^: 3373, 3076, 2229

### 3.3. Microwave assisted conversion of aldehydes into oximes under basic conditions

A 25 mL reaction flask was charged with a ground mixture of an aldehyde (1 mmol), Na_2_CO_3_ (5 mmol, 0.51 g) and hydroxylamine hydrochloride (5 mmol, 0.35g). The reaction mixture was placed at a CEM Discovery microwave reactor (power-time method) and irradiated at 100 W for 5 minutes, with air-cooling and magnetic stirring for the duraction. After cooling to room temperature, the reaction mixture was resuspended in CH_2_Cl_2_ (10 mL), filtered and a sample submitted to CG/MS analysis.

## 4. Conclusions

Titanium oxide is a useful reagent for the acid promoted conversion of EDG-substituted aldehydes to nitriles through a solvent free, microwave assisted procedure. On the other hand, neutralization of the reaction mixture by addition of NaOH or Na_2_CO_3_ proved to be an efficient method for obtaining the corresponding aldoximes in quantitative conversion rates.
